# Evaluation of Clinical Practice Guidelines on Timing and Onset of Labour in Gestational Diabetes Mellitus: A Scoping Review

**DOI:** 10.1111/1471-0528.70191

**Published:** 2026-02-22

**Authors:** Kathy Lynch, Amanda Poprzeczny, Jennifer Fereday, Tracy Humphrey

**Affiliations:** ^1^ College of Health Adelaide University Adelaide South Australia Australia; ^2^ The Robinson Research Institute and Department of Obstetrics and Gynaecology Adelaide University Adelaide Australia; ^3^ Department of Obstetrics and Gynaecology The Women's and Children's Hospital, Women's and Babies Division Adelaide Australia; ^4^ Rosemary Bryant AO Research Centre Adelaide Univeristy Adelaide Australia; ^5^ College of Nursing and Health Sciences, Flinders University Adelaide South Australia Australia

**Keywords:** clinical practice guidelines, decision aid, gestational diabetes mellitus, scoping review, shared decision making, timing and onset of labour or birth

## Abstract

**Background:**

Gestational diabetes mellitus (GDM) is the most common pregnancy complication globally, yet recommendations for the timing and onset of labour vary. Clinical practice guidelines (CPGs) aim to improve safety and quality by guiding decision making through evidence‐based recommendations.

**Objectives:**

Scoping review with embedded guideline appraisal to assess the consistency of recommendations regarding the timing and onset of labour for women with GDM and whether variation was associated with guideline quality.

**Search Strategy:**

Databases (MEDLINE, CINAHL, EBSCO, Scopus, Embase, JBI Guidelines) and websites of relevant professional organisations were searched.

**Selection Criteria:**

Guidelines and consensus statements published from 2015 to January 2025, available in English from high‐income countries, were included.

**Data Collection and Analysis:**

Guideline quality was assessed using the AGREE‐II tool, and recommendations were analysed descriptively.

**Main Results:**

Of 1422 records screened, 24 CPGs met the inclusion criteria. The quality of the included CPGs varied, with inconsistent applicability and stakeholder involvement (mean AGREE‐II domain scores 55%), developmental rigour (43%), and editorial independence (37%). On the basis of the AGREE‐II scores, only 3 CPGs were recommended for use without modifications. Even among higher quality guidelines, recommendations for the onset and timing of labour varied, from 37 to 40+ 6 weeks.

**Conclusions:**

Considerable variation exists with regard to the quality of CPGs and recommendations about the timing of birth for women with GDM. These findings highlight the importance of shared decision making and the need for further research to assist women and their maternity care providers in discussing optimal birth timing.

## 1 Introduction

Gestational diabetes mellitus (GDM), defined as hyperglycaemia first recognised during pregnancy, is now the most common pregnancy complication globally, with prevalence estimates ranging from 14% to 25% depending on the population and diagnostic criteria [1, 2, 3]. This rise has been driven by epidemiological factors such as increasing maternal age, higher rates of overweight and obesity, more sedentary lifestyles, and changes in diagnostic threshold [2, 4, 5]. Women with GDM are at increased risk of hypertensive disorders of pregnancy, induction of labour (IOL), and caesarean section. The infants born to women with GDM are more likely to be born large for gestational age (LGA), have neonatal hypoglycaemia, and require admission to the nursery [6, 7, 8].

Despite the frequency of GDM, there remains substantial uncertainty regarding optimal clinical management, particularly in relation to the timing and onset of labour. GDM is increasingly cited as a reason for IOL; however, the gestational age at which this does and should occur varies widely [9, 10, 11]. Robust evidence to guide these decisions is limited. Available studies are constrained by small sample sizes, non‐randomised designs, inconsistent diagnostic thresholds for GDM, and heterogeneous management protocols [6]. Notably, randomised trials comparing induction versus expectant management in GDM, such as the GINEXMAL trial, have not provided definitive guidance on optimal gestational age for planned birth, contributing to ongoing clinical uncertainty [12].

These limitations contribute to conflicting interpretation of maternal and fetal risk and consequently wide variation in practice. Current recommendations range from routine induction at 38 weeks to expectant management until 40+ 6 weeks [13, 14, 15]. The increase in IOL for women with GDM is suggested to be driven more by clinician interpretation, clinical caution, and inconsistent guidelines rather than by robust evidence [16], although other factors may also contribute.

Clinical practice guidelines (CPGs) aim to support safe, high‐quality and consistent care on the basis of the best available evidence [17]. In the context of GDM and labour timing and onset, limited and inconsistent evidence has translated into variation in guideline recommendations. This variability may expose women and infants to unnecessary interventions and associated risks, contribute to inconsistent care, and place additional strains on health care systems.

To date, the methodological quality of CPGs and their recommendations regarding the timing and onset of labour in GDM have not been systematically evaluated. Although variation in GDM guideline recommendations has been reported previously [18], existing work has largely described variability in general terms rather than examining timing and onset of labour as a discrete and clinically consequential decision domain.

This study, therefore, aimed to evaluate international CPGs addressing the timing and onset of labour in GDM by comparing the consistency of recommendations and examining whether variation was related to guideline quality. Using a scoping review with embedded guideline appraisal, we identified relevant CPGs, appraised their development quality using the AGREE‐II instrument [19], and synthesised diagnostic criteria, gestational age thresholds for planned birth, recommendations for timing and onset of labour and key clinical considerations.

## 2 Methods

This review was conducted in accordance with the Preferred Reporting Items for Systematic Reviews and Meta‐Analysis Extension for Scoping reviews (PRISMA‐ScR) [20], and guided the Joanna Briggs Institute (JBI) methodology for scoping reviews [21]. A priori protocol was developed and published on Open Science Framework https://doi.org/10.17605/osf.io/7xje9.

### 2.1 Inclusion and Exclusion Criteria

CPGs published from 2015 onward were included, corresponding with the widespread adoption of the International Association of Diabetes in Pregnancy Study group (IADPS) diagnostic criteria [5] first introduced in 2010, endorsed by the World Health Organisation in 2013 and formally adopted in Australia in 2015. Guidelines were limited to English language publications issued by local, national and international bodies in the Organisation for Economic Co‐operation and Development (OECD) member countries, selected for their broadly comparable healthcare systems. Eligible guidelines included professional society guidelines, consensus statements and systematic reviews explicitly addressing diagnosis, management and timing and onset of labour for women with GDM.

Broader diabetes guidelines were included only where they contained recommendations that were clearly applicable to women with GDM. Institutional or local protocols were eligible if intended to function as formal clinical practice guidelines. Where multiple or overlapping versions were identified, only the most recent version was included.

Guidelines were excluded if published before 2015, did not include recommendations on labour timing, or focused solely on the management of pre‐existing diabetes.

### 2.2 Search Strategy and Data Sources

An initial search on MEDLINE (via Ovid) was developed in collaboration with an academic librarian, adapting the Canadian Agency for Drugs and Technologies in Health (CADTH) filter and incorporating relevant Medical Subject Headings (MeSH) and keywords.

This strategy was adapted and applied to the databases CINHAL (via EBSCOhost), Scopus, Embase (via Ovid) and JBI Evidenced‐Based Practice between 9/1/25 to 16/1/25. Recognising that CPGs are often not published in medical journals, a manual search using Google Scholar and Google was conducted between 21 and 26 January 2025, targeting websites of professional organisations and health authorities likely to publish relevant GDM guidelines. Additional documents were identified via professional networks. The complete search strategy is outlined in Appendix S1.

### 2.3 Data Extraction

All references were imported into Endnote [22] and then uploaded into Covidence [23] where duplicates were removed. Two reviewers (KL and AP) independently screened titles and abstracts, followed by full text review for eligibility. Discrepancies were resolved by a third reviewer (JF).

Data were extracted independently by two researchers (KL and AP) using a custom data extraction form in Covidence. The form was pilot tested and refined as needed. Extracted data included guideline characteristics (publication date, country, issuing body, methodology development method), method of screening and diagnosis of GDM, specific recommendations regarding timing and onset of labour, clinical variables (such as insulin use or unstable glycaemic control) impacting this recommendation and other relevant considerations.

### 2.4 Guideline Quality Appraisal

Methodological quality was assessed using the AGREE‐II instrument [19], which comprises 23 items across six independent domains (scope and purpose, stakeholder involvement, rigour of development, clarity and presentation, applicability, and editorial independence). Each item was scored on a 7‐point scale (1 = strongly disagree to 7 = strongly agree).

Two reviewers (KL and AP) independently appraised each guideline. Discrepancies of two or more points were resolved through discussion and consensus; otherwise, the mean of the two raters for each item was used. Domain scores were calculated according to AGREE‐II guidance [19]. An overall guideline quality score was also assigned (1–7 scale) with higher scores indicating that more of the criteria were met.

Guidelines were recommended for use in clinical practice on the basis of thresholds adopted in similar reviews [24, 25, 26]. Specifically, guidelines were recommended if most domain scores exceeded 60%, recommended with modifications if domain scores ranged between 30% and 60%. As the objective of this study was to evaluate the evidence underpinning recommendations on the timing and onset of labour, particular emphasis was placed on the ‘Rigour of Development’ domain. Guidelines were not recommended for use in practice if this domain scored below 30%, regardless of scores in the other domains.

### 2.5 Data Analysis

Guideline recommendations were mapped against the AGREE‐II scores to explore potential associations between methodological quality and clinical guidance. Extracted data included GDM diagnostic criteria, recommendations on the timing and type of labour onset (including gestational age thresholds) and considerations for specific clinical scenarios (e.g., insulin use or unstable glycaemic control). The analysis specifically examined whether variations in recommendations could be explained by differences in ‘Rigour of Development’ scores and overall AGREE −11 scores.

## 3 Results

**FIGURE 1:**
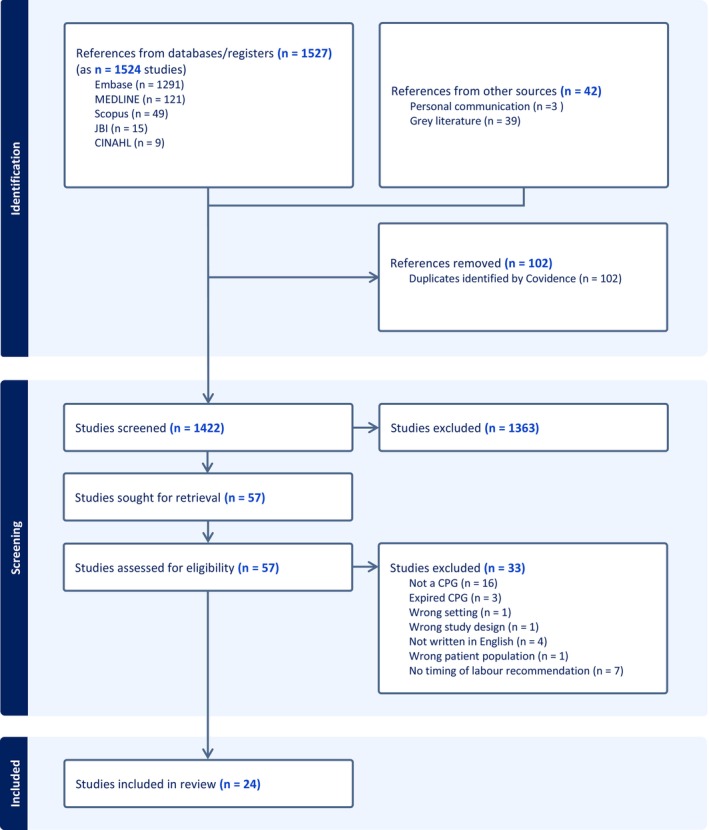
Timing and onset of labour for women with gestational diabetes mellitus.

A total of 1484 publications were identified through electronic database searches, with an additional 42 retrieved from grey literature and professional communications. Of the 57 articles screened at full text level, 24 met the selection criteria (Figure 1).

### 3.1 Guideline Characteristics

The characteristics and methodological development processes of the included CPGs are summarised in Table 2.

The included guidelines originated from Australia (*n* = 8; 33%) [13, 27, 28, 29, 30, 31, 32, 33], the United Kingdom (*n* = 5; 20%) [15, 34, 35, 36, 37], the United States (*n* = 4; 17%) [38, 39, 40, 41] and Europe (*n* = 3; 13%) [42, 43, 44]. One guideline was published by Japan [45], Canada [14] New Zealand [46] and an International collaborative group [47].

Just over half of the CPGs (*n* = 15; 63%) were developed at the state, regional or local health service level [13, 27, 28, 29, 30, 31, 32, 33, 34, 35, 36, 37, 39, 41, 46], typically by multidisciplinary teams. Six guidelines (25%) were developed at the national level by obstetric, gynaecology or diabetes societies [14, 15, 38, 42, 44, 45]. Two (8%) were consensus statements issued by obstetric and gynaecology societies [43, 45] and one guideline formed part of a global framework for GDM management [47].

### 3.2 Guidelines Methodological Approaches

Most CPGs (*n* = 17; 71%) used evidence to inform their recommendations. Among these, seven (29%) [14, 15, 37, 38, 42, 45, 47] applied formal evidence grading systems such as GRADE or SIGN, whereas the remainder cited supporting evidence without indicating the quality or strength. One CPG did not specify its development methodology [46].

Guidelines developed through consensus [27, 28, 29] or expert opinion [30, 31] typically involved multidisciplinary working groups and were designed as practice protocols for local use. One CPG [40] combined an evidence‐based review using the GRADE with expert opinion, presented in narrative format.

### 3.3 Screening and Diagnostic Criteria

Recommendations for screening and diagnosing GDM varied considerably. The IADPSG criteria were most commonly used, adopted by 12 (50%) guidelines [13, 27, 28, 29, 31, 32, 33, 41, 42, 43, 44, 47]. Four guidelines (17%) applied the National Institute for Health and Care Excellence (NICE) criteria [15, 34, 35, 36], and another four (17%) used a two‐step approach involving a 50 g glucose challenge followed by a 75 g or 100 g oral glucose tolerance test [14, 38, 39, 45] Two guidelines (8%) applied diagnostic thresholds between IADPSG and NICE criteria [37, 46], and one did not specify the diagnostic criteria used.

### 3.4 Timing and Onset of Labour

Across the 24 included CPGs, recommendations regarding the timing and onset of labour for women with GDM varied significantly (Table 1). Most guidelines (*n* = 22; 92%) differentiated recommendations on the basis of glycaemic control and treatment modality (diet, metformin, or insulin), although gestational age thresholds differed in clinically meaningful ways.

**TABLE 1:** Summary of guidelines on the timing and onset of labour for women with GDM.

Title	Publication year	Country guideline jurisdiction	Issuing body	Methodology used for grading the strength of recommendations	Methodology used to reach recommendations	GDM classification	Timing of labour recommendations	Timing of labour recommendations	Timing of labour recommendations	Considerations
							Diet controlled	Insulin controlled	Poorly controlled	
Guidelines for obstetrical practice in Japan: Japan Society of Obstetrics and Gynaecology and Japan Association of Obstetricians and Gynaecologists 2020 edition [1]	2022	Japan National	Japan Society of Obstetrics and Gynaecology and the Japan Association of Obstetricians and Gynaecologist	Japan‐specific approach developed by JSOG and JAOG	Evidence‐based, but consensus when evidence was weak	2 step (50 g–75 g load)	Consider expectant management, but also IOL from 37 weeks. IOL with consideration of cervical maturation	Not stated	After 37 weeks consider on an individual basis	Consider fetal growth, cervical assessment, glycaemic control
Diabetes in pregnancy includes pre‐existing and gestational diabetes [2]	2023	Australia State/Territory	Canberra Health services	Not stated	Evidence‐based	IADPSG	41 + 3	39–40	38–39	Consider fetal growth, maternal comorbidities and glycaemic control
Gestational diabetes mellitus (GDM) [3]	2022	Australia State/Territory	Queensland Health	Not stated	Evidence‐based	IADPSG	Expectant	39	Not Stated	If suspected fetal macrosomia or other complications, consider birth from 38 + 0–39 + 0 weeks gestation
Diabetes [4]	2022	Australia State/Territory	Government of Western Australia North Metropolitan Health Service	Not stated	Consensus	IADPSG	40	38–39	Not stated	El LSCS if EFW > 4250 g
Gestational diabetes [5]	2018	Australia State/Territory	Department of Health Victoria	Not stated	Evidence‐based	IADPSG	Expectant management	38: consider on the basis of glycaemic control	Individualised on the basis of clinical presentation	Defines Suboptimal glycaemic control: three or more fasting BGLs ≥ 5.0 mmol in the preceding week or three or more two‐hour postprandial BGLs ≥ 6.7 mmol/L in the preceding week
Management of GDM [6]	2024	Australia Local	South Eastern Sydney Local Health District	Not stated	Evidence‐based	IADPSG	Expectant management	Expectant if well controlled, but if glucose above target or suboptimal engagement IOL at 40 weeks	40 weeks if on high dose insulin (> 0.5 units/kg current weight), or at 40–41 weeks if on low dose insulin (< 0.5 units/kg current weight)	Consider fetal growth, maternal comorbidities, glycaemic control
Maternity‐Diabetes in Pregnancy, Labour, Birth & the Postnatal Period [7]	2018	Australia State/Territory	NSW Health Hunter New England Local Health District	Not stated	Consensus	IADPSG	40–41	39	Not stated	
Management of GDM in Pregnancy [8]	2023	Australia Local	Tasmanian health service‐ northwest regional hospital	Not stated	Expert Opinion	IADPSG	Expectant management	40 if on insulin < 30 units 38–39 if on > 30 us of insulin	Not stated	
Management of Gestational Diabetes [9]	2024	Australia Local	Tasmanian Health Service WACS	Not stated	Expert opinion	Fasting > 5.3–6.9 mmol, 1 h > 10.6, 2 h 9–11	Expectant management	39 weeks if on “higher doses, 40 if on minimum” insulin	Not stated	“Induction earlier than 39 weeks should only occur for other complications in addition to insulin and after consultation with O&G Consultant”
Diabetes and Pregnancy [10]	2018	Canada National	Diabetes Canada	Diabetes Canada evidence grading system	Evidence‐based	2‐step classification 50 g and 75 g load	38–40	Not stated	Not stated specifically, “Earlier or later IOL considered on the basis of glycaemic control and absence of other comorbidities”	
Clinical practice guidelines on diabetes mellitus and pregnancy: ΙI. GDM [11]	2020	Greece National	Hellenic Endocrine society, Hellenic society of maternal‐fetal medicine	Not stated	Evidence‐based	IADPSG	40–41	39–39 + 6	Not stated	
GDM, diagnostics, therapy and follow‐up care [12]	2023	Germany National	German diabetes association	AWMF Association of Scientific Medical Societies in Germany	Evidence‐based	IADPS	39–39 + 6	40	“Do not consider before 38 weeks due to associated morbidity with preterm birth; instead, optimise BGLs”	“An induction in the 39th +0–39th +6 week may be considered but is associated with a 50% increase in the rate of inducing and does not reduce neonatal morbidity”
Guideline on Pregnancy and Diabetes by the Society of Specialists in Perinatology (PUDER) Turkey [13]	2020	Turkey National	Pregnancy and Diabetes workshop of PUDER	Not stated	Consensus	IADPSG However, also state can have 2 step according to preferences of clinician	39–39 + 6	38–39 + 6	36–38 + 6	
The International Federation of Gynaecology and Obstetrics (FIGO) Initiative on GDM: A pragmatic guide for diagnosis, management, and care [14]	2015	International	The International Federation of Gynaecology and Obstetrics	GRADE	Evidence‐based	IADPSG	40–41	Not stated	38–39	
Antenatal outpatient care for gestational diabetes [15]	2022	New Zealand Local	Canterbury District Health Board	Not stated	Not stated	75 g OGTT FBG ≥ 5.5 mmol/L OR 2 h BG ≥ 9.0 mmol/L	41	40	Not stated	
Diabetes in pregnancy: management from preconception to the postnatal period [16]	2020	UK National	National Institute for Health and Care Excellence	GRADE	Evidence‐based	NICE definition	40 + 6	40 + 6	Not stated	“Consider elective birth before 40 weeks plus 6 days for women with GDM who have fetal or maternal complications”
SIGN 171 Management of diabetes in pregnancy [17]	2024	UK National	NHS Scotland	SIGN System	Evidence‐based	Single step 75 g OGTT‐ diagnosed if fasting glucose ≥ 5.3 mmol/L (one‐hour post 75 g oral glucose load ≥ 10.6 mmol/L, where used), two‐hour post 75 g oral glucose load ≥ 9.0 mmol/L.	40 + 6	40 + 6	Not stated	“Consider elective birth before 40 weeks for women with gestational diabetes if there are maternal or fetal complications”
Guideline for the Management of Diabetes in Pregnancy [18]	2022	UK National	NHS Wales	Not stated	Evidence‐based	Nice Definition	40	“Consideration given for earlier delivery (than 40 weeks) for women with GDM who are managed with metformin or insulin or any complications, as for preexisting diabetes type 1 or 2”	Not stated	
Guideline for the Management of Diabetes in Pregnancy [19]	2023	UK Local	NHS Barnsley Hospital	Not stated	Evidence‐based	NICE definition	40 + 6	38–40 if on oral medication 38–39 if on insulin	Consider at 38–39	
GDM [20]	2022	UK Local	NHS University Hospitals of Leicester	Not stated	Evidence‐based	Nice definition	40 + 6	40	Not stated	“Timing should include individualised risk assessment including recent USS including growth velocity and liquor volume”
ACOG Practice Bulletin No. 190 GDM [21]	2018	United states National	The American College of Obstetricians and Gynaecologists	US Preventive Services Task Force	Evidence‐based	2 Step classification. Screening 1 h 50 g and then 100 g load if screened high according to institutions threshold	40 + 6	39–39 + 6	37–38 + 8	
Diabetes and Pregnancy Program [22]	2017	United States Local	OHSU Center for Women's Health	Not stated	Evidence‐based	IADPSG	39–41	39	36–38	Need to balance “the maternal and fetal risks of advancing gestation versus the neonatal risks for earlier delivery must be evaluated as diabetes poses ongoing risks to the pregnancy”
Gestational Diabetes Screening and Treatment Guideline [23]	2024	United States Local	Kaiser Foundation Health Plan of Washington	Not stated	Evidence‐based	2‐step classification 50 g and 75 g load	40–40 + 6	39–39 + 6	38–38 + 6	
GDM: obstetric issues and management [24]	2023	United States	UpToDate group Local	Some personal recommendations, others on the basis of GRADE	Expert opinion	Not stated‐ reviewed in a separate document	39–41	39	If on insulin 37–38 + 6, if on diet 39	

Abbreviations: BGLs, blood glucose levels; EFW, estimated fetal weight; GDM, gestational diabetes mellitus; IADPSG, International Association of Diabetes and Pregnancy Study Groups; IOL, induction of labour; LSCS, lower segment caesarean section; OGTT, oral glucose tolerance test; USS, ultrasound scan.

#### 3.4.1 Diet‐Controlled GDM

For women with diet‐controlled GDM, recommendations ranged from early‐term induction to expectant management beyond term. The most common approach, recommended by 13 CPGs (54%) was expectant management or birth at 4O+ 6 weeks or later [13, 15, 29, 30, 31, 32, 33, 34, 36, 37, 38, 45, 46]. Five CPGs (21%) recommended earlier planned birth from between 38 or 39 weeks [14, 40, 41, 42, 43].

Overall, the recommended timing of birth for diet‐controlled GDM varied by up to 3 weeks across guidelines.

#### 3.4.2 Metformin or Insulin GDM

Greater variation was observed among recommendations for women requiring pharmacological treatment. 19 (79%) of the CPGS recommended planned birth between 38 and 40 weeks [13, 27, 28, 29, 30, 31, 32, 33, 34, 35, 36, 38, 39, 40, 41, 42, 43, 44, 46], three recommended birth between 40 and 40+ 6 weeks and three didn't address this at all.

Some guidelines treated pharmacological treatment as a threshold for earlier birth, recommending birth from 38 to 39 weeks, irrespective of insulin dose or degree of control [27, 28, 33, 43, 48]. Others adopted a more nuanced approach, differentiating recommendations on the basis of insulin dose or level of glycaemic control, with recommended timing ranging from 39 weeks to 41 weeks [30, 31, 34, 35].

#### 3.4.3 Poor Glycaemic Control

The majority (54%) of CPGs did not provide specific recommendations for women with poorly controlled GDM [14, 15, 27, 28, 30, 31, 32, 33, 35, 36, 37, 44, 46]. Among those that did, approaches varied, with some basing recommendations on insulin dose [29, 35, 40], and others (33%) suggesting timing of birth between gestations from 36 to 38 + 6, typically framed as an individualised decision informed by fetal growth, maternal comorbidities, and persistent hyperglycaemia [13, 34, 38, 39, 41, 43, 45, 47].

### 3.5 Additional Considerations

Several CPGs identified additional factors influencing decisions about the optimal timing and onset of labour. Five guidelines (*n* = 21%) referenced fetal growth [13, 29, 33, 36, 45], four (17%) considered the presence of maternal comorbidities [13, 15, 29, 37], and six (25%) emphasised the degree of glycaemic control [13, 14, 29, 32, 33, 45] as a determinant of timing. Many CPGs recommended individualised care on the basis of clinical findings, with three (13%) specifically advising shared decision making with women [34, 36, 37] and one providing specific guidance for management when a woman declined IOL [27].

In summary, the 24 CPGs revealed significant geographical variation in GDM management approaches regarding timing and onset of labour recommendations. UK guidelines were the most consistent, with most recommending birth at 40+ 6 weeks for women with diet‐controlled GDM [15, 34, 36, 37]. US guidelines, using mixed diagnostic criteria, recommended the earliest intervention globally (37–39+ 6 for women using insulin) [38, 39, 40, 41]. Australian guidelines, although predominantly applying IADPSG criteria, showed the greatest variation in timing of birth recommendations and provided the most detailed insulin dose‐based guidance [13, 27, 28, 29, 30, 31, 32, 33]. European CPGs consistently used IADPGS criteria and generally recommended more moderate timing (38–40 weeks for women using insulin) [42, 43, 44].

### 3.6 Methodological Quality

#### 3.6.1 Overall Assessment

The overall quality of the reviewed guidelines varied considerably, reflecting differences in the development processes. Mean AGREE‐II domain scores were: scope and purpose 94%, stakeholder involvement 55%, rigour of development 43%, clarity of presentation 88%, applicability 55%, editorial independence 37% (Table 2). Three guidelines were recommended for use [14, 15, 37], 10 were recommended for use with modifications [30, 33, 35, 39, 41, 42, 44, 45, 47, 48] and 11 were not recommended for use [13, 27, 28, 29, 31, 32, 34, 36, 40, 44, 46]. The mean of the six AGREE‐II domain scores across all guidelines was 58.7%.

**TABLE 2:** Guideline assessment according to the AGREE‐II instrument (*n* = 24).

Country/Region	Guidelines	Guideline jurisdiction	Scope and purpose (%)	Stakeholder involvement (%)	Rigour of development (%)	Clarity of presentation (%)	Applicability (%)	Editorial independence (%)	Mean domain scores (%)
Asia	Japan [1]	National	88	53	42	75	33	96	50
Australia	Australian Capital Territory‐Canberra [2]	State/Territory	100	33	27	97	35	25	50
	Queensland [3]	State/Territory	86	75	69	78	73	63	58
	Western Australia [4]	State/Territory	94	44	25	94	58	0	67
	Victoria [5]	State/Territory	86	19	20	89	50	8	58
	NSW South East [6]	Local	100	56	28	94	46	25	50
	NSW Hunter [7]	Local	100	61	10	83	65	12	67
	Tasmania North [8]	Local	100	75	30	86	58	13	50
	Tasmania North West [9]	Local	89	56	28	81	60	0	33
Canada	Canada [10]	National	89	47	65	89	75	83	83
Europe	Greece [11]	National	100	44	43	92	34	75	27
	Germany [12]	National	100	72	79	83	56	75	83
	Turkey [13]	National	75	36	17	81	23	83	33
International	Figo [14]	International	100	67	70	97	88	79	75
New Zealand	Canterbury [15]	Local	92	17	17	97	27	0	42
United Kingdom	NICE (England) [16]	National	100	100	98	83	98	92	100
	SIGN (Scotland) [17]	National	100	97	84	100	100	96	100
	NHS Wales [18]	National	100	64	31	89	60	0	58
	NHS Barnsley [19]	Local	100	58	24	92	60	0	50
	NHS Leicester [20]	Local	94	47	25	94	69	13	58
United States	ACOG [21]	National	94	42	64	100	52	33	75
	OHSU [22]	Local	100	52	36	83	50	0	67
	Kaiser Permanente [23]	Local	81	75	60	92	33	8	44
	UpToDate [24]	Local	89	31	52	83	25	50	33

Guidelines recommended for use with modifications commonly lacked detail regarding development methodology processes for formulating recommendations and strategies for implementation and updating. These omissions limited the transparency and reliability of the recommendations.

Nine guidelines [28, 29, 30, 31, 34, 36, 39, 41, 46] were developed as local or institutional protocols intended primarily for local implementation. Although often adapted from more rigorously developed state or national guidelines, these scored poorly because of limited documentation on development methods and justification for recommendations.

## 4 Discussion

### 4.1 Main Findings

This scoping review identified inconsistency in the quality, scope and breadth of recommendations across the CPGs addressing the timing and onset of labour for women with GDM. Many lacked transparency in evidence synthesis, editorial independence, and processes to ensure rigour, raising concerns about their overall trustworthiness.

### 4.2 Strengths and Limitations

Several limitations should be considered when interpreting these findings. Only English language CPGs from OECD countries were included, which may introduce bias and limit generalisability. Broader diabetes guidelines containing relevant recommendations for GDM may have been overlooked, and CPGs not available online or published in alternative formats may not have been captured. Furthermore, the limited availability of high‐quality trials restricts the robustness of recommendations, highlighting the need for further research.

### 4.3 Interpretation

Given our focus on the ‘Rigour of Development’ domain, the review departs from AGREE‐II guidance, which advises equal weighting of all domains. Prioritising rigour nevertheless provided insight into whether methodological quality influenced recommendations. To our knowledge, this is the first scoping review to apply AGREE‐II in this context, explicitly examining the relationship between rigour and guideline recommendations. Although only three CPGs were recommended without modification, most achieved a score of 50% or more, suggesting a reasonable baseline. Common areas for improvement included greater detail in development processes and clearer reporting of editorial independence.

Importantly, higher rigour scores did not correlate with greater consistency in recommendations. Even among the most methodologically robust CPGs, substantial variation persisted. This suggests that factors beyond methodological quality, such as local clinical norms, expert consensus and differing interpretations of the evidence, may shape recommendations. Differences in resource availability, population health needs and cultural contexts may also contribute, particularly where diagnostic methods and criteria for GDM vary. Similar inconsistencies have been reported in other broader maternity care guidelines [16, 49, 50], suggesting these issues are not unique to GDM but reflect wider challenges in maternity guideline development.

This interpretive divergence was most evident for women with diet‐controlled GDM, where the recommended timing of birth ranged from induction at 38 weeks to expectant management beyond 40 + 6 weeks. This three‐week variation reflects differing interpretations of the same limited evidence base rather than clear differences in guideline quality. For example, the German national guideline [42] advises caution when offering induction before 40 weeks, explicitly noting no reduction in neonatal morbidity and increased intervention rates. In contrast, Diabetes Canada [14] supports offering induction from 38 weeks to potentially reduce stillbirth, while acknowledging this recommendation is largely consensus‐based and supported by low‐certainty evidence. These contrasting positions illustrate how the same evidence base can lead to markedly different clinical thresholds.

Variation was also pronounced for women requiring pharmacological treatment. Some CPGs treated insulin or oral hypoglycaemic therapy as a threshold for earlier birth, recommending birth from 38 to 39 weeks irrespective of dose or degree of glycaemic control. Others adopted a more nuanced approach, stratifying recommendations by insulin dose or control, with suggested timing extending to 41 weeks. This lack of consensus reflects ongoing uncertainty regarding the balance of maternal, fetal, and neonatal risks in the absence of robust comparative data.

### 4.4 Implications for Clinical Practice and Research

The implications are significant. CPGs are intended to enhance the quality and safety of maternity care by guiding clinicians to use the best available evidence [17]. However, variability may complicate decision making, risk inconsistent or poor‐quality care, and may undermine women's confidence in clinical advice. For example, although a diagnosis of GDM alone is not a recognised clinical indication for IOL [10], some CPGs implied otherwise.

Emerging evidence indicates that increasing rates of early‐term birth, often driven by elective induction or caesarean section, are associated with higher rates of neonatal respiratory morbidity, infant hospital admissions for infection, and potential impacts on child development, whereas planned birth at 39–40 weeks does not appear to carry the same level of risk [51]. Ecological analyses across Europe further suggest that higher early‐term birth rates may be linked with lower stillbirth rates, although overall perinatal mortality did not differ. These findings highlight the complexity of balancing potential benefits against the well‐documented risks of early term birth, suggesting that even small differences in gestational age thresholds, such as those observed across the CPGS for women with GDM, may have meaningful implications for neonatal and child health.

Variability in recommendations also affects service planning, influencing induction rates, caesarean workload and resource allocation. Future research should clarify optimal timing of birth for women with GDM, examine how guideline variation shapes service delivery and women's experiences, and ensure women's perspectives help inform guideline development.

In the meantime, clinicians should navigate discordant guidance through shared decision making, presenting uncertainties transparently and aligning decisions with women's values. Internationally, women‐centred models of care are increasingly emphasised [52, 53, 54, 55, 56] and in Australia, national safety and quality standards highlight the importance of supporting women to make informed choices [57]. However, only two of the reviewed CPGs incorporated women's perspectives in their development, and few addressed care pathways for women who decline IOL, highlighting a persistent gap in women‐centred care.

Decision aids may help address this gap. A Cochrane review of 209 trials [58] demonstrates that decision aids improve knowledge, clarify values and foster more active participation in care. Although underused in maternity care, early research suggests decision aids for IOL can strengthen communication and support informed decision making [59].

For women with GDM, decision aids may be particularly valuable in discussions about the timing and onset of labour, supporting care that aligns with women's values and preferences in an area where evidence remains limited, and guideline recommendations are inconsistent.

## 5 Conclusion

CPGs from English‐speaking OECD countries show considerable variation in recommendations regarding the timing and onset of labour for women with GDM. This variability persists even among guidelines of comparable quality, suggesting that it reflects differing interpretations of limited evidence rather than methodological rigour alone. Such inconsistency highlights the challenges of translating evidence into clinical practice. Clinicians should remain aware of this variability and prioritise shared decision making within a woman‐centred framework. Decision aids may offer a valuable tool to support informed, collaborative discussions, helping align practice with women's values while navigating the challenges posed by inconsistent guideline recommendations.

## Author Contributions

Kathy Lynch conceived and designed the study, conducted the literature search, and led the scoping review. She developed and piloted the data extraction form in Covidence, undertook title and abstract screening, full‐text review, and guideline quality appraisal using the AGREE II instrument. Kathy further analysed and interpreted the data and drafted the manuscript. Amanda Poprzeczny contributed to title and abstract screening, full‐text review, data extraction, and quality assessment using the AGREE II instrument. Jennifer Fereday assisted with resolving discrepancies during screening, contributed to the interpretation of findings, and critically revised the manuscript. Tracy Humphrey contributed to the interpretation of results and provided substantive input during manuscript revision. All authors made substantial contributions to the conception or design of the work, the acquisition, analysis, or interpretation of data, drafting or critically revising the manuscript and have approved the final version for publication, and all agree to be accountable for the integrity and accuracy of the work.

## Funding

The authors have nothing to report.

## Conflicts of Interest

The authors declare no conflicts of interest.

## Supporting information


**Appendix S1:** Database search strategy.

## Data Availability

Data sharing is not applicable to this article as no datasets were generated or analysed during the current study.
