# Supplementary material for: Evaluation of Clinical Practice Guidelines on Timing and Onset of Labour in Gestational Diabetes Mellitus: A Scoping Review
Source: BJOG. 2026 Feb 22;133(7):1342–57. doi: 10.1111/1471-0528.70191 (PMC13143554; doi:10.1111/1471-0528.70191)
Supplement: Supplementary file 1 — Appendix S1: Database search strategy. [file BJO-133-1342-s001.docx]

Appendix S1

Data base search strategy

**Medline**

**9-1-25**-

Diabetes, Gestational/
(gestational adj2 diabetes).ti,ab,kf.
"pregnancy induced diabetes".ti,ab,kf.
GDM.ti,ab,kf.
(hyperglyc* adj2 pregnan*).ti,ab,kf.
"diabetes in pregnancy".ti,ab,kf.
(gestational hyperglycemia or gestational hyperglycaemia).ti,ab,kf.
or/1-7
exp Labor, Obstetric/
exp Delivery, Obstetric/
near term.ti,ab,kf.
post term.ti,ab,kf.
Obstetrics/
obstetrics.ti,ab,kf.
(planned adj3 birth).ti,ab,kf.
(spontaneous adj2 onset).ti,ab,kf.
(elective adj2 birth).ti,ab,kf.
(labor or labour or induce* or induction or delivery or birth or episiotom* or caesarean* or cesarean* or IOL).ti,ab,kf.
or/9-18
clinical protocols/
consensus/
exp consensus development conference/
exp consensus development conferences as topic/
critical pathways/
exp guideline/
guidelines as topic/
practice guideline/
practice guidelines as topic/
health planning guidelines/
Clinical Decision Rules/
(position statement* or policy statement* or practice parameter* or best practice*).ti,ab,kf.
(standards or guideline or guidelines).ti,kf.
((practice or treatment* or clinical) adj guideline*).ab.
(CPG or CPGs).ti.
consensus*.ti,kf.
consensus*.ab. /freq=2
((critical or clinical or practice) adj2 (path or paths or pathway or pathways or protocol*)).ti,ab,kf.
recommendat*.ti,kf. or guideline recommendation*.ab.
(care adj2 (standard or path or paths or pathway or pathways or map or maps or plan or plans)).ti,ab,kf.
(guideline* or standards or consensus* or recommendat*).au.
(algorithm* adj2 (screening or examination or test or tested or testing or assessment* or diagnosis or diagnoses or diagnosed or diagnosing)).ti,ab,kf.
(algorithm* adj2 (pharmacotherap* or chemotherap* or chemotreatment* or therap* or treatment* or intervention*)).ti,ab,kf.
Clinical Protocols/
(clinical adj2 guid*).ti,ab,kf.
(practice adj2 guid*).ti,ab,kf.
(clinical adj2 decision*).ti,ab,kf.
or/20-46
8 and 19 and 47
limit 48 to yr="2015 -Current"
(initiative on gestational diabetes mellitus: a pragmatic guide for diagnosis, management and care).m_titl.
49 and 50

**Scopus**

**16-1/25**

( TITLE-ABS-KEY

( clinical AND pathway OR clinical AND protocol OR consensus OR consensus AND development OR clinical AND pathway OR practice AND guideline OR clinical AND decision AND rule OR( position AND statement* OR policy AND statement* OR practice AND parameter* OR best AND practice* ) OR ( standards OR guideline OR guidelines ) ( ( practice OR treatment* OR clinical ) guideline* ) OR ( cpg OR cpgs ) OR consensus* AND consensus* OR ( ( critical OR clinical OR practice ) W/2 ( path OR paths OR pathway OR pathways OR protocol* ) ) OR recommendat* OR guideline AND recommendation* ) )

AND ( TITLE-ABS-KEY

( gestational AND diabetes ) OR ( pregnancy AND diabetes AND mellitus ) OR gestational AND diabetes OR "pregnancy induced diabetes" OR ( hyperglyc* W/2 pregnan* ) OR "diabetes in pregnancy" OR ( gestational AND hyperglycemia OR gestational AND hyperglycaemia ) )

AND ( TITLE-ABS-KEY ( labor OR obstetric AND delivery OR cesarean AND section OR near AND term OR post AND term OR obstetrics OR ( planned W/3 birth ) ) OR ( spontaneous W/2 onset ) OR ( elective W/2 birth ) OR ( labor OR labour OR induce* OR induction OR delivery OR birth OR episiotom* OR caesarean* OR cesarean* ) ) AND PUBYEAR > 2014 AND PUBYEAR < 2025 AND ( LIMIT-TO ( LANGUAGE , "English" ) )

**Embase**

**9-1-25**

gestational diabetes/
Pregnancy diabetes mellitus/
gestational diabetes mellitus/
exp labor/
exp obstetric delivery/
exp cesarean section/
near term.ti,ab,kf.
post term.ti,ab,kf.
(planned adj3 birth).ti,ab,kf.
(spontaneous adj2 onset).ti,ab,kf.
(elective adj2 birth).ti,ab,kf.
(labor or labour or induce* or induction or delivery or birth or episiotom* or caesarean* or cesarean* or IOL).ti,ab,kf.
practice guideline/
guideline$.ti,ab,kf.
consensus.ti,ab,kf.
position statement$.ti,ab,kf.
exp health care policy/ or exp policy/
recommendation$.ti,ab,kf.
1 or 2 or 3
4 or 5 or 6
13 or 14 or 15 or 16 or 17 or 18
19 and 20 and 21
limit 22 to yr="2015 -Current"
4 or 5 or 6 or 7 or 8 or 9 or 10 or 11 or 12
19 and 21 and 23 and 24

**JBI**

**9-1-25**

clinical practice guidelines.mp. [mp=text, heading word, subject area node word, title]
guidelines.mp. [mp=text, heading word, subject area node word, title]
clinical pathways.mp. [mp=text, heading word, subject area node word, title]
clinical decision rules.mp. [mp=text, heading word, subject area node word, title]
nursing practice evidence based.mp. [mp=text, heading word, subject area node word, title]
medical practice evidence based.mp. [mp=text, heading word, subject area node word, title]
midwifery practice.mp. [mp=text, heading word, subject area node word, title]
gestational diabetes.mp. [mp=text, heading word, subject area node word, title]
(labour and birth planning).mp. [mp=text, heading word, subject area node word, title]
spontaneous labour.mp. [mp=text, heading word, subject area node word, title]
cesarean section.mp. [mp=text, heading word, subject area node word, title]
planned birth.mp. [mp=text, heading word, subject area node word, title]
spontaneous birth.mp. [mp=text, heading word, subject area node word, title]
obstetric care.mp. [mp=text, heading word, subject area node word, title]
midwifery care.mp. [mp=text, heading word, subject area node word, title]
post term birth.mp. [mp=text, heading word, subject area node word, title]
1 or 2 or 3 or 4 or 5 or 6 or 7
9 or 10 or 11 or 12 or 13 or 14 or 15 or 16
8 and 17 and 18
limit 19 to yr="2015 -Current"

**CINAHL**

**9-1-25**

(MH (Diabetes, Gestational + OR "pregnancy induced diabetes" OR GDM OR "diabetes in pregnancy"))

( MH (Labor, Obstetric + OR “Delivery, Obstetric OR near term OR post term OR Obstetrics OR planned birth) OR (spontaneous adj2 onset) OR (elective adj2 birth) OR (labor or labour or induce* or induction or delivery or birth or episiotom* or caesarean* or cesarean* or IOL)

(MH(practice or treatment* or clinical) adj guideline*) OR (CPG or CPGs) OR consensus* OR ((critical or clinical or practice) adj2 (path or paths or pathway or pathways or protocol*)) OR recommendat* OR guideline recommendation* OR
(care adj2 (standard or path or paths or pathway or pathways or map or maps or plan or plans)) OR
(guideline* or standards or consensus* or recommendat*) OR (screening or examination or test or tested or testing or assessment* or diagnosis or diagnoses or diagnosed or diagnosing)) OR (MH (Clinical Protocols OR (clinical adj2 guid*)) OR (practice adj2 guid*) OR (clinical adj2 decision*)

Grey literature search

21-26-1-25

National Sources

- Websites of all state and territory health departments in Australia
- Federal government health department website
- Professional organizations:
  - Australian College of Midwives (ACM)
  - The Royal Australian and New Zealand College of Obstetricians and Gynaecologists (RANZCOG)
  - Australasian Diabetes in Pregnancy Society (ADIPS)

International Sources

- Canadian Practice Guidelines
- National Institute for Health and Clinical Excellence (NICE)
- Guidelines International Network
- Scottish Intercollegiate Guidelines Network (SIGN)
- New Zealand Guidelines Group
- National Health and Medical Research Council
- American College of Obstetricians and Gynaecologists
- American Diabetes Association
- the Society of Obstetricians and Gynaecologists of Canada (SOGC)
- Royal College of Obstetricians and Gynaecologists (RCOG)
